# ICBP90 belongs to a new family of proteins with an expression that is deregulated in cancer cells

**DOI:** 10.1038/sj.bjc.6601068

**Published:** 2003-07-01

**Authors:** M Mousli, R Hopfner, A-Q Abbady, D Monté, M Jeanblanc, P Oudet, B Louis, C Bronner

**Affiliations:** 1 Inserm U392, Faculté de Pharmacie, 74 route du Rhin, B.P. 60024, 67401 Illkirch Cedex, France; 2 IGBMC, CNRS UMR 7104, Inserm U184, B.P. 163, 67404 Illkirch, Cedex, France; 3 Institut de Biologie de Lille, UMR 8117 CNRS/Institut Pasteur de Lille, 1 rue Calmette, 59021 Lille Cedex, France; 4 Centre de Pathologie, 18 rue Kempf, 67000 Strasbourg, France

**Keywords:** ICBP90, topoisomerase IIα, cancer, cell cycle, E2F, retinoblastoma protein

## Abstract

ICBP90 (Inverted CCAAT box Binding Protein of 90 kDa) is a recently identified nuclear protein that binds to one of the inverted CCAAT boxes of the topoisomerase II*α* (TopoII*α*) gene promoter. Here, we show that ICBP90 shares structural homology with several other proteins, including Np95, the human and mouse NIRF, suggesting the emergence of a new family of nuclear proteins. Towards elucidating the functions of this family, we analysed the expression of ICBP90 in various cancer or noncancer cell lines and in normal or breast carcinoma tissues. We found that cancer cell lines express higher levels of ICBP90 and TopoII*α* than noncancer cell lines. By using cell-cycle phase-blocking drugs, we show that in primary cultured human lung fibroblasts, ICBP90 expression peaks at late G1 and during G2/M phases. In contrast, cancer cell lines such as HeLa, Jurkat and A549 show constant ICBP90 expression throughout the entire cell cycle. The effect of overexpression of E2F-1 is more efficient on ICBP90 and TopoII*α* expression in noncancer cells (IMR90, WI38) than in cancer cells (U2OS, SaOs). Together, these results show that ICBP90 expression is altered in cancer cell lines and is upregulated by E2F-1 overexpression with an efficiency depending on the cancer status of the cell line.

DNA-topoisomerase II*α* (TopoII*α*) is the primary target of several anticancer drugs currently used in cancer treatments (reviewed in Wang, 1996; Withoff *et al*, 1996). TopoII*α* expression correlates with drug sensitivity. For instance, enhanced sensitivity to anti-TopoII drugs was observed after adenovirus-mediated *TopoIIα* gene transfer in a human breast cancer cell line (Zhou *et al*, 2001). There is a marked heterogeneity of TopoII*α* expression within different breast cancers (Sandri *et al*, 1996), various tissues (Turley *et al*, 1997; Withoff *et al*, 1999) and cell lines (Mo *et al*, 1998), which is responsible for the wide variety of anti-TopoII drug sensitivity. TopoII*α* expression is linked to cell growth and is cell-cycle regulated (reviewed in Isaacs *et al*, 1998; Nitiss, 1998). E2F-1 has been reported to regulate TopoII*α* expression and cell chemosensitivity to anti-TopoII*α* drugs (Hofland *et al*, 2000; Kalma *et al*, 2001; Dong *et al*, 2002). However, the *TopoIIα* gene promoter lacks an E2F-consensus binding site considering the first 700 nucleotides from the transcription start site (Hochhauser *et al*, 1992), suggesting the involvement of an intermediary actor in the *TopoIIα* gene regulation by E2F members.

Using the one hybrid system, we have identified a novel human protein able to bind the inverted CCAAT box 2 (ICB2) of the *TopoIIα* gene promoter and that we named ICBP90 (Inverted CCAAT box Binding Protein of 90 kDa) (Hopfner *et al*, 2000). The ICB2 is known to play a crucial role in the *TopoIIα* gene expression regulation (Isaacs *et al*, 1996; Tolner *et al*, 2001; reviewed in Bronner *et al*, 2002). We have also recently shown that transient overexpression of ICBP90 could overcome cell contact inhibition by forcing TopoII*α* expression in primary cultures of human lung fibroblasts (Hopfner *et al*, 2002). Interestingly, the promoter of the *ICBP90* gene contains an E2F-consensus binding site close to one of the transcription start sites (Hopfner *et al*, 2001). The E2F-1 is a member of the E2F family that is found to be elevated in cancer cells (Lemass *et al*, 1998; Suzuki *et al*, 1999).

Consequently, to further elucidate the role of ICBP90, we hypothesised that ICBP90 may be an intermediary actor in the E2F-induced TopoII*α* expression, and that its expression may be deregulated in cancer cells *vs* noncancer cells. For this, we investigated (i) whether ICBP90 shares structural features with other proteins exhibiting known properties; (ii) the expression of ICBP90, TopoII*α* and pRB in various cell lines; (iii) the effects of E2F-1 overexpression on TopoII*α* and ICBP90 expression; (iv) the cell-cycle-dependent expression of ICBP90 in normal cells *vs* cancer cells; and (v) the ICBP90 expression in primary breast carcinoma tissue *vs* noncancer breast tissue.

## MATERIALS AND METHODS

### Materials

The mouse monoclonal antibody (mAb) directed against ICBP90 (1RC1C-10) was engineered in our laboratory by a standard method (Brou *et al*, 1993). The anti-TopoIIα mAb (Ki-S1), alkaline phosphatase-conjugated anti-mouse and alkaline phosphatase-conjugated anti-rabbit antibodies were purchased from Roche Diagnostics (Mannheim, Germany). The anti-E2F-1 mAb was obtained from BD Biosciences Clontech (Palo Alto, CA, USA). The rabbit antiactin antibody, aphidicolin, L-mimosine, nocodazole and propidium iodide were obtained from Sigma Chemicals (St Louis, MO, USA) and the mouse anti-pRB (retinoblastoma protein) mAb MAB3186 from Chemicon International (Temecula, CA, USA). Tween®20, nitro blue tetrazolium/5-bromo-4-chloro-3-indolyl-phosphate (NBT/BCIP) stock solution and protease inhibitor cocktail were from Roche Diagnostics.

### Cell cultures and cell synchronisation

Human lung fibroblasts in primary culture were prepared and cultured as described elsewhere (Hopfner *et al*, 2000). The human cell lines A549, Jurkat, MCF7, HeLa, 293, WI-38, IMR-90, U2OS, SaOS and MDA were obtained from the American Type Culture Collection (Manassas, VA, USA) and were grown in DMEM supplemented with 10% FCS, 2 mM glutamine, 100 U ml^−1^ penicillin and 50 *μ*g ml^−1^ streptomycin.

To synchronise fibroblasts, HeLa, A549 and Jurkat cells were treated with L-mimosine (100 *μ*g ml^−1^), aphidicolin (1 *μ*g ml^−1^) or nocodazole (50 ng ml^−1^) for 20 h to arrest cells in the G1 phase, the S phase or in the G2/M phases, respectively (Cheng and Kuchta, 1993; Vasquez *et al*, 1997; Wang *et al*, 2000). Cells were released from cell-cycle blocks by washing with phosphate-buffered saline (PBS) at pH 7.4, and fresh medium was added.

### Adenoviral infections

The recombinant adenovirus AdCMV-Flag-E2F1 was obtained by insertion of the human E2F-1 coding sequence into the pAdCMV-Flag vector, followed by homologous recombination in *Escherichia coli* (Blais *et al*, 2002). The FLAG epitope (MAYKDDDKL) was appended at the N-terminus of the coding sequence of human E2F-1 to allow easy detection of transgene expression by Western blot. Details of cloning are available upon request. Viral stocks were produced as previously described (Blais *et al*, 2002) and viral titres were determined by a plaque assay in 293 cells and defined as PFU ml^−1^. Cells were then infected by adding virus stocks directly to the culture medium at an input multiplicity ranging between 100 and 300 viral particles per cell. The infected cells were harvested 18 h later and total proteins were extracted for immunodetection as described (Blais *et al*, 2002).

### Flow cytometry

Cell suspensions were fixed by incubation for 15 min on ice with 5% formaldehyde in PBS, followed by resuspension in 100% ethanol and storage at −20°C for 16 h. DNA labelling was obtained by incubation for 10 h at 20°C in darkness with 50 *μ*g ml^−1^ of propidium iodide and 50 *μ*g ml^−1^ of RNAse A. Experiments were performed with at least 10 000 cells. The propidium iodide fluorescence was quantified with a FACScan flow cytometer and data were analysed with the CellQuest software (Becton Dickinson, Franklin Lakes, NJ, USA).

### Western blotting

Whole cell extract preparations are described elsewhere (Hopfner *et al*, 2000; Blais *et al*, 2002). Blots were probed with either the 1RC1C-10 mAb (0.5 *μ*g ml^−1^), the Ki-S1 mAb (0.1 *μ*g ml^−1^), the anti-pRB (2 *μ*g ml^−1^), the anti-E2F-1 (4 *μ*g ml^−1^), or the antiactin antibody (0.8 *μ*g ml^−1^). As secondary antibodies, an alkaline phosphatase-conjugated anti-mouse antibody or, for the antiactin antibody, an alkaline phosphatase-conjugated anti-rabbit antibody was used at 200 mU ml^−1^. Signals were detected with an NBT/BCIP stock solution according to the instructions of the manufacturer. Protein bands were quantified on scanned blots with the NIH Image 1.62 software (National Institutes of Health, Bethesda, MD, USA).

### Immunohistochemistry

Indirect immunoperoxidase staining of ICBP90 in breast tissues was carried out as described elsewhere (Hopfner *et al*, 2000). Briefly, specimens from noncancer breast tissue, high-grade or low-grade primary breast carcinomas were embedded in paraffin and fixed in 10% buffered formalin (Sigma). Histologic sections (3 *μ*m) were incubated overnight at room temperature with the 1RC1C-10 mAb, and specifically bound antibodies were visualised by a streptavidin–biotin complex (Dako LSAB2 System Kit, DAKO, Carpinteria, CA, USA).

## RESULTS

### Identification of a new family of proteins involved in cell-cycle regulation

**Figure 1:**
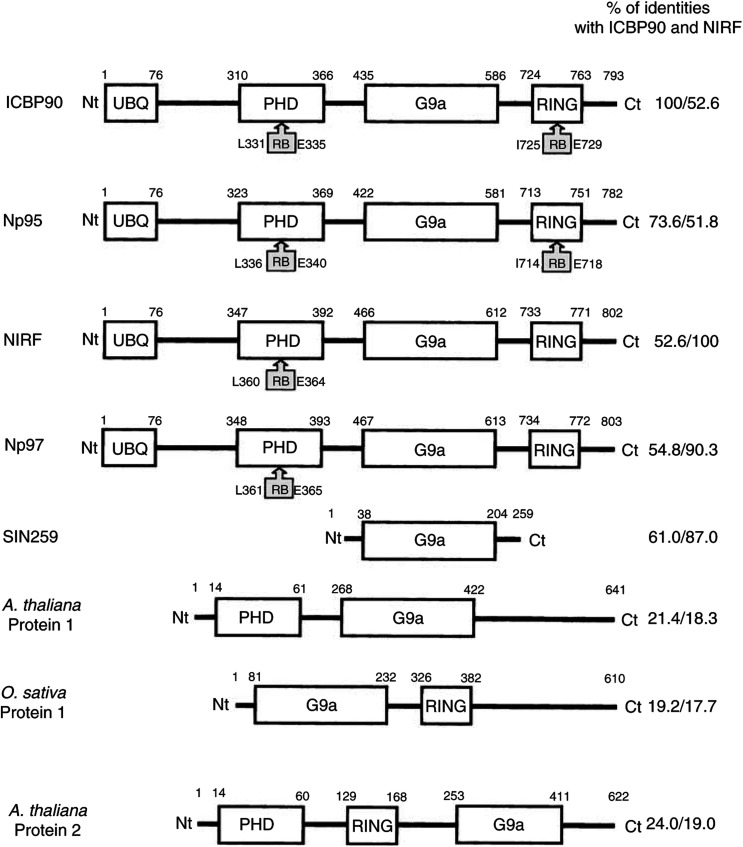
Structural features of ICBP90, mouse Np95, human Np97 (NIRF), mouse Np97, two *A. thaliana* proteins and an *O. sativa* protein. The lengths of the ubiquitin-like domain (UBQ), the PHD finger domain (C3HC4-type), the G9a domain (G9a), the RING finger (C4HC3-type) are delimited by the numbered letters corresponding to the positions of the amino acids. The retinoblastoma protein binding sites (RB) are indicated by the presence of hatched boxes with the positioned amino acids. Information on published data or through Genbank (http://www.ncbi.nlm.nih.gov/) are indicated in the ‘Results’ section. Values at the C terminal (Ct) of the proteins indicate percentages of identity to ICBP90 and NIRF, respectively, on the length of the concerned proteins. The beginning of the proteins is indicated by Nt (N-terminal part of the proteins). SIN259 means Similar to ICBP90 and NIRF of 259 amino-acids length.

The primary structure alignments of eight proteins, that is, ICBP90 (Hopfner *et al*, 2000), Np95 (Fujimori *et al*, 1998), NIRF (NP95/ICBP90 Ring Finger, Mori *et al*, 2002), Np97 (mouse NIRF, Genbank accession number BAB68317), a human 259-amino-acid-long protein similar to ICBP90 (Genbank accession number XM_066969), two *Arabidopsis thaliana* proteins 1 and 2 (Genbank accession numbers AAG29238 and NP_176778, respectively) and an *Oryza sativa* protein (Genbank accession number AAG03103) are shown in Figure 1

. ICBP90 has several structural domains, including a ubiquitin-like domain, a G9a domain and two zinc-finger domains. All these features are, so far, only found in Np95 and mouse or human NIRF. The mouse NIRF exhibits 54.8% of identity with ICBP90 and 52.6% with the human NIRF. The human and mouse NIRF share 90.3% identity with each other, whereas the homology between ICBP90 and Np95 only reaches 73.4%. A short human protein of 259 amino acids, which we called SIN259 (for *S*imilar to *I*CBP90 and *N*IRF), has also been identified, sharing 61.0% identity with ICBP90 and 87% with human NIRF. ICBP90 and NIRF exhibit 21.4 and 18.3% identity with the *A. thaliana* protein 1, respectively. Also, ICBP90 and NIRF show 19.2 and 17.7% identity with an *O. sativa* protein, respectively. The *A. thaliana* protein lacks the ubiquitin-like domain as well as the RING finger domain, while the *O. sativa* protein lacks the ubiquitin-like domain as well as the PHD finger domain. It is interesting to mention that the two plant proteins together contain all the structural domains of the three mammalian proteins, except the ubiquitin-like domain that can be considered as dispensable for the activity of these proteins. These similarities would suggest that NIRF and ICBP90 derive from two common ancestral genes, provided that an equivalent of the *O. sativa* protein exists in *A. thaliana.*

A second *A. thaliana* protein sharing 24.0 and 19% identity with ICBP90 and NIRF, respectively, possesses a PHD, a RING finger as well as a G9a domain, but not in the order found in ICBP90, therefore questioning its membership to this family.

### ICBP90, TopoIIα and pRB expression in normal and cancer cell lines

**Figure 2:**
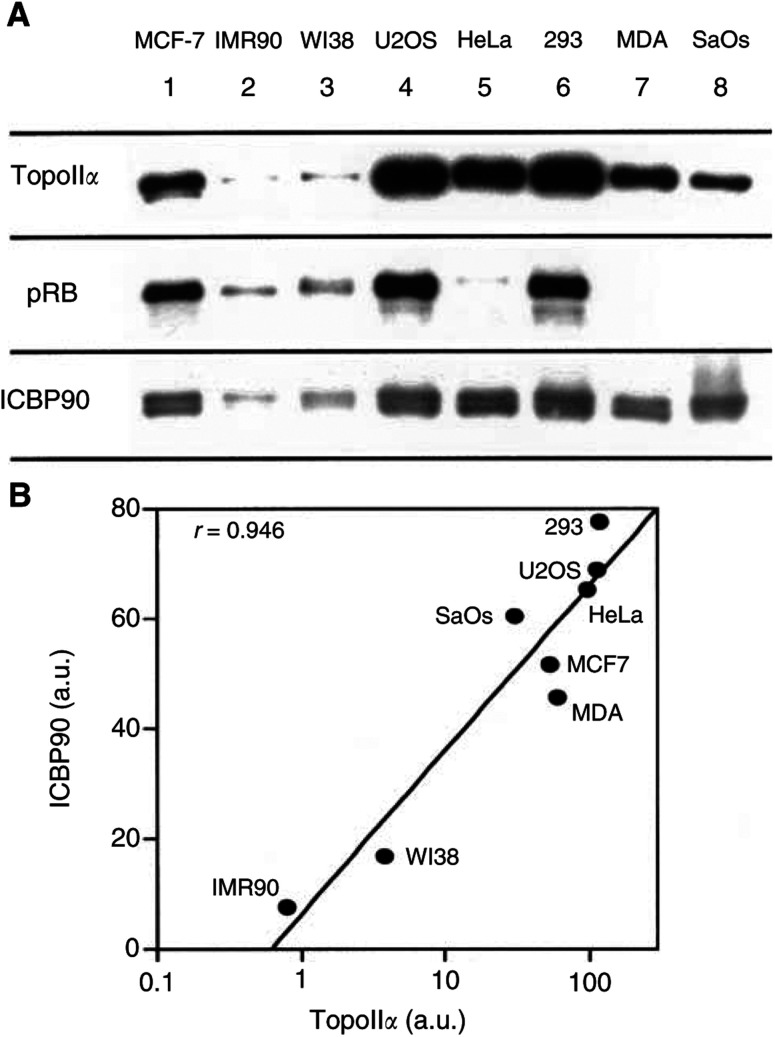
Expression of TopoII*α*, pRB and ICBP90 in normal and cancer cell lines. (**A**) Western blot analysis of TopoII*α*, pRB and ICBP90 expression in different cancer cell lines and noncancer cell lines. Lanes 1–8 contain whole cell extracts from MCF-7, IMR90, WI38 U2OS, HeLa, 293, MDA468 and SaOS cells, respectively. For all the cell lines, cells were collected under proliferating conditions, that is, between 60 and 70% confluence. Labelling with the corresponding monoclonal antibodies is described in ‘Materials and Methods’. Data are representative of two separate experiments. (**B**) Correlation between ICBP90 and TopoII*α* expression was quantified and expressed as arbitrary units with the NIH 1.62 image software by scanning the Western blot bands.

ICBP90, pRB and TopoII*α* expression was investigated in MCF-7 (a breast cancer cell line), IMR90 (human embryo lung fibroblasts), WI38 (human embryo lung fibroblasts), U2OS (an osteosarcoma cell line), HeLa (a cervix cancer cell line), 293 (human kidney embryonic cells), MDA468 (a breast cancer cell line) and SaOS cells (an osteosarcoma cell line) (Figure 2A

). Cancer cell lines show high levels of ICBP90 and TopoII*α* expression. This is the case of MCF-7 (lane 1), U2OS (lane 4), HeLa cells (lane 5), MDA (lane 7) and SaOS (lane 8), and also of 293 cells (lane 6) that are noncancer cells. In contrast, human lung fibroblasts such as IMR90 (lane 2) and WI38 (lane 3) cells exhibit low levels of ICBP90 and TopoII*α* expression when compared to the previous cancer cell lines. A very good correlation was found between TopoII*α* and ICBP90 expression (Figure 2B), suggesting that ICBP90 contributes to the elevation of TopoII*α* expression in cancer cells. U2OS followed by 293 cells and MCF-7 cells are the cell lines that express high levels of pRB. IMR90 and WI38 cells express low levels of pRB. A slight expression of pRB was observed in HeLa cells but it corresponds to the hyperphosphorylated (inactive) form of pRB as the band was slightly higher. MDA and SaOS did not express pRB, but they were chosen in order to see if they expressed the highest levels of TopoII*α* since in these cell lines pRB cannot exert inhibitory effects on its cellular targets, for example, E2F-1 and putatively ICBP90. Unexpectedly, these cell lines expressed less TopoII*α* than U2OS or 293 cell lines that showed the highest pRB expressions (Figure 2A).

### Effect of E2F-1 overexpression on ICBP90 and TopoIIα expression in various cell lines

**Figure 3:**
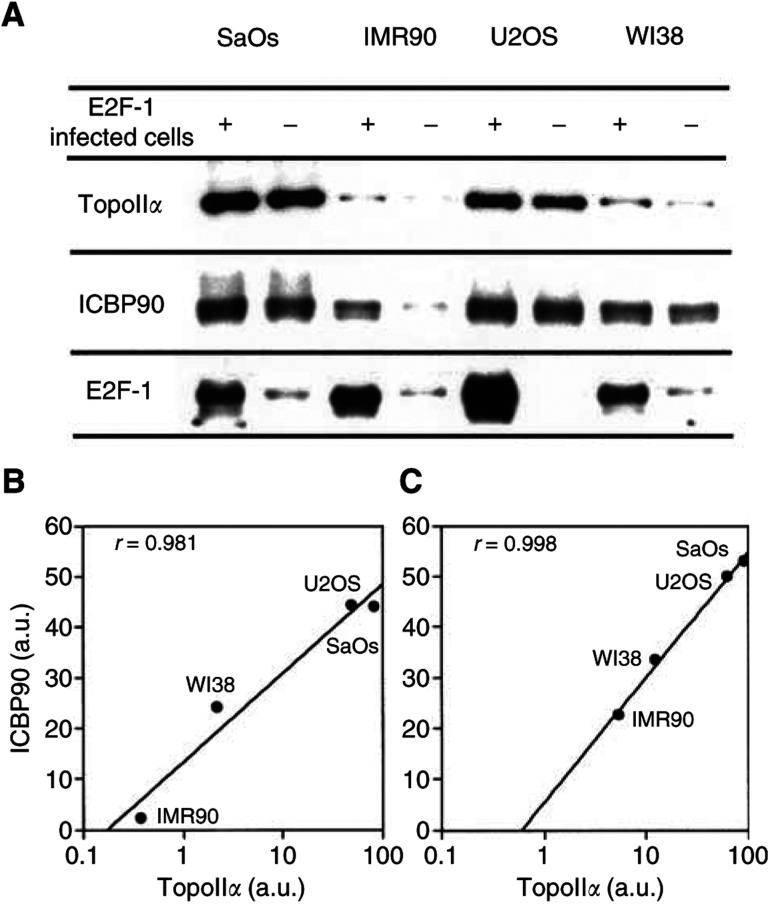
Effects of overexpression of E2F-1 on TopoII*α* and ICBP90 in different cell lines. Infection and Western blot were carried out as described in ‘Materials and Methods’. Lanes correspond to SaOs (1, 2), IMR90 (3, 4), U2OS (5, 6) and WI38 (7, 8) cells transfected with E2F-1 or not, respectively, as indicated on the first line. For all cell lines, cells were collected under proliferating conditions, that is, between 60 and 70% confluence. Data are representative of two separate experiments. (**B**) Correlation was made with the four cell lines that do not overexpress E2F-1. (**C**) Correlation was made with the four cell lines that overexpress E2F-1.

Two cancer (SaOs and U2OS) and two noncancer (IMR90 and WI38) cell lines were tested for E2F-1 overexpression experiments (Figure 3

). The E2F-1-adenoviral infection of these cell lines led to 7.1-, 12.5- and 10.2-fold increases of E2F-1 expression in SaOS, IMR90 and WI38 cells, respectively. A strong increase of E2F-1 expression in infected U20S cells was observed but could not be calculated, as basal E2F-1 expression was not detectable in our experimental conditions. The order of efficiency of the E2F-1 over-expression on ICBP90 expression was IMR90, WI38, SaOs and U2OS with increases of 990.8, 38.1, 20.9 and 13.3%, respectively. The order of efficiency of the E2F-1 overexpression on the TopoII*α* expression was IMR90, WI38, U2OS and SaOs with increases of 1445.9, 552.5, 26.7 and 13.3%. These results show that overexpression of E2F-1 using adenoviral infection experiments enhances ICBP90 and TopoII*α* expressions with various efficiencies according to the cell type. Also, these results show that the efficiency of the E2F-1 overexpression on the ICBP90 and TopoII*α* expression is higher in noncancer cell lines (IMR90 and WI38 cells) than in cancer cell lines (SaOs and U2OS). There is always a good correlation between ICBP90 and TopoII*α* expression either in noninfected cells (Figure 3B) or in E2F-1 adenoviral infected cells (Figure 3C).

### Analysis of ICBP90 expression in noncancer human lung fibroblasts during the cell cycle

**Figure 4:**
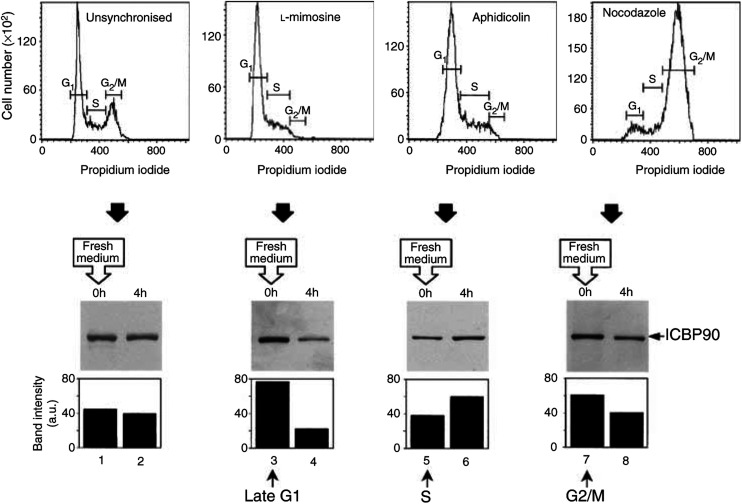
Cell-cycle expression of ICBP90 in human lung fibroblasts. Fibroblasts were synchronised in late G1 phase, S phase and G2/M phases with cell-cycle-blocking drugs L-mimosine (100 *μ*g ml^−1^), aphidicolin (1 *μ*g ml^−1^) and nocodazole (50 ng ml^−1^), respectively. Cell-cycle profiles of synchronised cells were determined by flow cytometry (upper panels). ICBP90 levels were analysed in cell-cycle-blocked fibroblasts (lower panels, lanes 1, 3, 5 and 7) and then determined 4 h after addition of fresh medium without drug (lower panels, lanes 2, 4, 6 and 8). Protein bands were quantified on scanned blots with the NIH Image 1.62 software. Column graphs indicate protein amount for each band in a.u. Data are representative of three independent experiments.

To study the cell cycle of ICBP90 expression in noncancer cells, primary cultured human lung fibroblasts cells were synchronised with reversible cell-cycle-blocking drugs (Figure 4

). In unsynchronised fibroblasts, 65, 15 and 20% of the cells were found in G0/G1, S and G2/M phases, respectively (Figure 4). As a control in the absence of drug, the addition of fresh medium decreased the ICBP90 level from 45.0 arbitrary units (a.u.) (lane 1) to 40.0 a.u. (lane 2). In L-mimosine-treated fibroblasts, 88% of the cells were in G1 phase while 12% of the cells remained in S and G2/M phases, and ICBP90 expression increased from 45.0 a.u. (lane 1) to 76.7 a.u. (lane 3). After 4 h, ICBP90 levels decreased to 22.2 a.u. (lane 4), suggesting that ICBP90 expression decreases as cells enter S phase. Using the S phase-blocking drug aphidicolin, the percentage of cells in S phase reached 65%, while 35% of the cells were still in G1 and no cells remained in the G2/M phases. Arrest by aphidicolin resulted in ICBP90 levels of 37.9 a.u. (lane 5), which increased to 60.8 a.u. 4 h after drug release (lane 6), indicating an enhancement of ICBP90 as cells leave the S phase. In nocodazole-synchronised fibroblasts, 92% of the cells were in G2/M phases. The ICBP90 levels reached 62.8 a.u. in G2/M-blocked cells (lane 7), and decreased to 40.8 a.u. 4 h after drug release, as cells re-enter the G1 phase (lane 8). Together, these results show that ICBP90 levels are maximum in L-mimosine- and nocodazole-treated human lung fibroblasts. Similar results were obtained with human tracheal smooth muscle cells, whereas aphidicolin and nocodazole were without any effect on the ICBP90 expression in HeLa cells (data not shown).

### Expression of ICBP90 in lung fibroblasts and HeLa cells synchronised with L-mimosine

Figure 5

**Figure 5:**
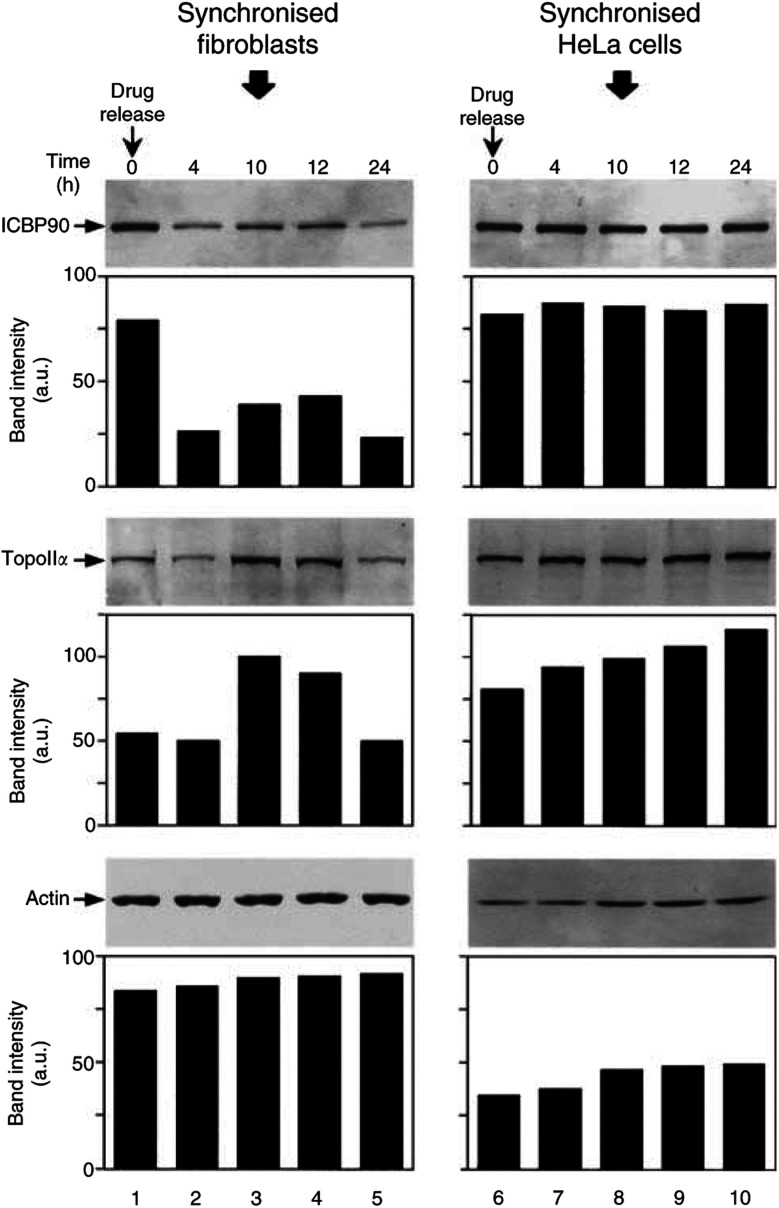
ICBP90 expression is cell-cycle-regulated in noncancer human lung fibroblasts, but not in the HeLa cell line. Lung fibroblasts and HeLa cells were synchronised at late G1 with L-mimosine at 100 *μ*g ml^−1^. Cells were washed with PBS to remove L-mimosine (Drug release), then fresh medium was added and the synchronised cells were incubated for 24 h. At indicated times, synchronised fibroblasts (lanes 1–5) and synchronised HeLa cells (lanes 6–10) were collected and used to prepare cell lysates that were analysed by Western blot to determine ICBP90, TopoII*α* and actin levels. Protein bands were analysed on scanned blots with the NIH Image 1.62 software. Column graphs indicate protein amounts in a.u. Data are representative of four separate experiments.

shows the cell-cycle expression of ICBP90 and TopoII*α* in human lung fibroblasts and HeLa cells. Synchronisation of the human lung fibroblasts at late G1 by L-mimosine conducted to ICBP90 and TopoII*α* expressions of 78.4 and 53.8 a.u., respectively (lane 1). After 4 h of drug release, the ICBP90 level decreased to 25.8 a.u., whereas the TopoII*α* level remained stable at 41.0 a.u. (lane 2). However, after 10 h of incubation, ICBP90 levels increased to 38.8 a.u. and TopoII*α* expression reached its highest level of 112.0 a.u., (lane 3). After 12 h of incubation, a slight increase in ICBP90 and a decrease in TopoII*α* expression occurred (lane 4). After 24 h, ICBP90 and TopoII*α* levels decreased to 22.8 and 44.3 a.u., respectively (lane 5). In L-mimosine-synchronised HeLa cells, an ICBP90 level of 82.1 a.u. was observed (lane 6), similar to that observed for fibroblasts (lane 1), and no significant variation was observed after L-mimosine release. Indeed, the ICBP90 protein level was stable at 84.6±2.1 a.u. from 4 to 24 h of incubation (lanes 7–10). For these late G1-arrested cells, a high TopoII*α* protein level of 80.2 a.u. was observed (lane 6). The TopoII*α* level increased progressively after release of L-mimosine from 93, 98, 105 to 115 a.u. after 4, 8, 12 and 24 h of incubation, respectively (lanes 7–10). This was not surprising, as TopoII*α* expression is known to maintain its cell-cycle-dependent regulation in cancer cells, notably in HeLa cells (Isaacs *et al*, 1998). A similar cell-cycle-independent expression of ICBP90 was obtained with several other cancer cell lines including the A549 cell line and the Jurkat T cell line (data not shown).

### Expression of ICBP90 in breast carcinoma tissues *vs* normal breast tissue

**Figure 6:**
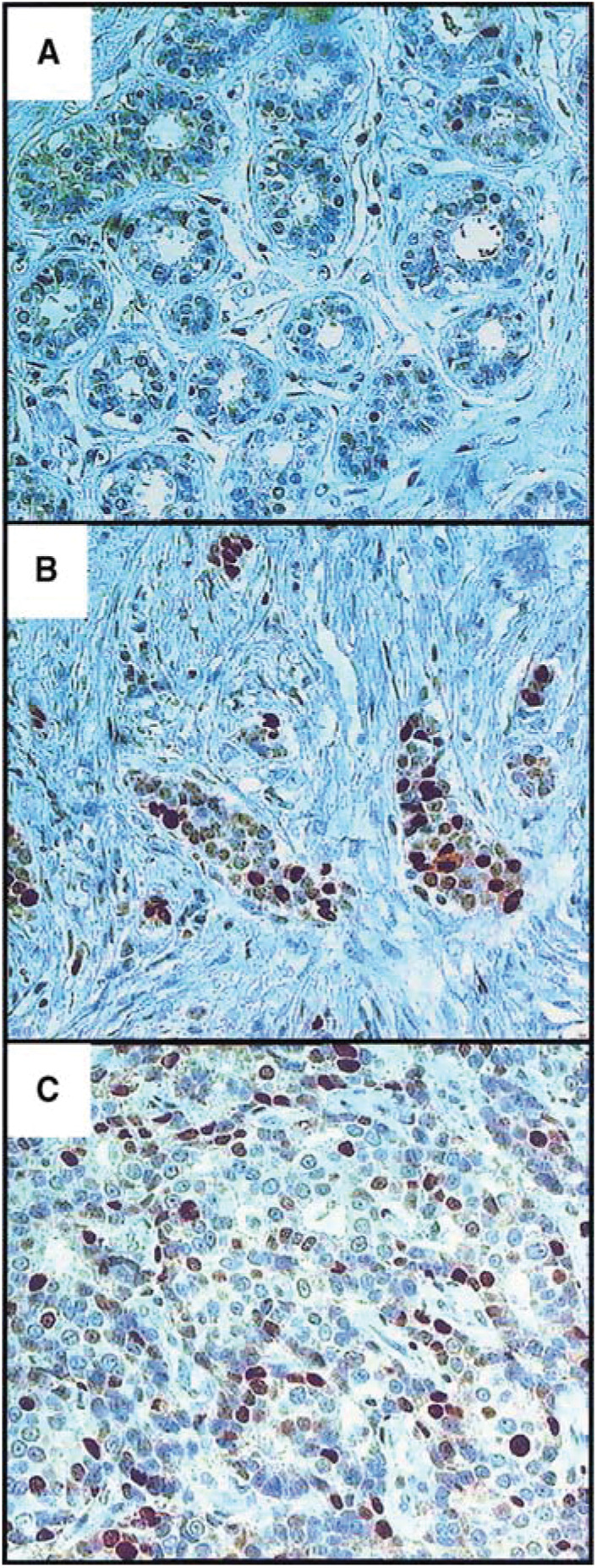
ICBP90 expression in normal breast tissue and breast carcinomas. Immunoperoxidase staining was carried out on histological sections in normal breast tissue (**A**) and in low-grade (**B**) or high-grade breast carcinomas (**C**) with the anti-ICBP90 mAb (0.2 *μ*g ml^−1^). Original magnification: × 400.

The expression of ICBP90 was investigated in low-grade or high-grade primary breast carcinomas compared to normal breast tissue (Figure 6

). The percentages of ICBP90-labelled nuclei were <1, 5 and 15% for normal breast tissue (Figure 6A), low-grade (Figure 6B) and high-grade carcinomas (Figure 6C), respectively. These results show that cells expressing ICBP90 were very low in normal breast tissue, whereas in breast carcinomas the percentage of ICBP90-positive cells appears to follow the grade.

## DISCUSSION

We have recently identified a new protein able to bind, *in vitro*, the ICB2 element of the human *TopoIIα* gene promoter (Hopfner *et al*, 2000). Proteins shown in Figure 1 appear to belong to the same family of proteins involved in cell proliferation. The function of these proteins is not yet known, except for ICBP90, which is a putative regulator of the TopoII*α* gene expression (Hopfner *et al*, 2000,^2002^). They share several structural domains including two zinc finger domains (one of the PHD finger type and one of the RING finger type) and a G9a domain of unknown function. Interestingly, ICBP90, Np95 and the mouse and human NIRF share the Rb-binding site located in the PHD finger, but that located in the RING finger is present only in ICBP90 and Np95. This suggests that NIRF may have distinguished regulatory mechanism. Nevertheless, with 73.6% of identity we cannot yet conclude that Np95 is the mouse counterpart of ICBP90 since the identity between the mouse and human NIRF is 90.3%, a percentage that is usually expected for interspecies homologies between transcription factors. Since the *Np95* gene appears to participate in the stability of the genome (Muto *et al*, 2002), it is not excluded that ICBP90 and/or the aligned proteins exhibit features of tumour suppressor genes. Altogether these proteins may constitute a new family of transcription factors involved in cell proliferation.

ICBP90 peaks at late G1 and during G2/M phases in human lung fibroblasts. Since the increase in ICBP90 expression is maximum in late G1, we propose that ICBP90 is the transcription factor participating in the *TopoIIα* gene induction at the G1/S boundary. Such suggestion is consistent with the lack of E2F-binding sites in the *TopoIIα*gene promoter (Hochhauser *et al*, 1992) known to be important for induction of genes at the G1 to S transition (reviewed in Müller and Helin, 2000). TopoII*α* expression correlates well with ICBP90 expression after E2F-1 overexpression, suggesting that ICBP90 is the intermediary in the E2F-1-induced TopoII*α* gene expression. However, we do not exclude other possibilities such as the eviction of an Rb-associated repressor complex from the *TopoIIα* gene promoter by E2F-1. The transition from G1 to S phase is regulated by phosphorylation of the retinoblastoma protein (pRB) that is mediated by cyclin-dependent kinases (reviewed in Kaelin, 1999). The hypophosphorylated form of pRB interacts with transcription factors, for example and by this way counteracts their transcriptional activity. Considering the presence of consensus pRB-binding sites in ICBP90, we expected to find the highest TopoII*α* expression in pRB-null cells. Unfortunately, this was not the case questioning, at least in these cancer cell lines, whether ICBP90-dependent TopoII*α* expression is sensitive to pRB regulation.

Higher expression of ICBP90 was found in cancer cell lines (SaOs, MCF-7, U2OS, HeLa, MDA468) than noncancer cell lines (WI38, IMR90). The cell line 293 (an embryonic kidney cell line) appears to be an exception, but foetal kidney is among the ICBP90 mRNA richest tissues (Hopfner *et al*, 2000) as is the case for NIRF (Mori *et al*, 2002). This high expression of ICBP90 correlates with high levels of TopoII*α*, presenting ICBP90 as being involved in the overexpression of TopoII*α* in cancer cells (reviewed in Withoff *et al*, 1996; Isaacs *et al*, 1998). This higher expression may come from the lack of downregulation at confluence and/or during certain phases of the cell cycle. Indeed, we have previously shown that ICBP90 is still expressed in HeLa cells at confluence, whereas it is not expressed in confluent human fibroblasts (Hopfner *et al*, 2000). Furthermore, in the present study, we showed that the ICBP90 cell-cycle-dependent expression disappeared in HeLa, Jurkat cells and A549 cells. Np95 expression was shown to be upregulated in the Sphase and downregulated in G2/M phases in normal mouse T cells, but this variation was abolished in mouse tumour T cells (Muto *et al*, 1995). Also, human NIRF was shown to be still expressed in cancer cell lines at confluence but not in normal fibroblasts such as WI38 (Mori *et al*, 2002). Therefore, we propose that cell-cycle-deregulated expression of the members of this family of proteins is a new marker of malignity for cancer cell identification. Interestingly, we found that normal breast tissue was almost negative for ICBP90 staining, whereas a relation seems to take shape between the number of ICBP90-positive cells and the grade.

We found that E2F-1 overexpression increases TopoII*α* expression and ICBP90 expression, suggesting that this latter is an intermediary candidate in the E2F-1-mediated *TopoIIα* gene regulation (Hofland *et al*, 2000). The increase is moderate in cancer cells, such as SaOs cells, whereas it is highly efficient in noncancer cells, for example in the IMR90 cell line, probably due to the fact that ICBP90 is already expressed at a high level before E2F-1 overexpression. Therefore, it could explain why the E2F-1 overexpression is less efficient in SaOs cells than other cell lines in terms of sensitisation to anti-TopoII*α* drugs (Yang *et al*, 2001; Dong *et al*, 2002). Since these cells are pRB and p53 null cells, it is likely that the basal expression of E2F-1 is sufficient to induce submaximal ICBP90 and TopoII*α* expression, which would consequently minimize the effect of an E2F-1 overexpression on the cell sensitivity to anti-TopoII drugs (Yang *et al*, 2001). Furthermore, we believe that the deregulation of the ICBP90 expression in cancer cells involves E2F considering that high E2F activity has been documented in colon tumours and gastric carcinomas (Lemass *et al*, 1998; Suzuki *et al*, 1999), that an E2F binding site exits in the promoter of the ICBP90 gene (Hopfner *et al*, 2001) and that overexpression of E2F-1 has little effect on ICBP90 expression in cancer cell lines *vs* noncancer cell lines (present results).

In conclusion, we propose that ICBP90 belongs to a new family of proteins whose role remains to be further characterised and whose deregulated expression is a common feature of cancer cells that can be useful for cancer diagnosis.
